# Genomics‐Guided Efficient Identification of 2,5‐Diketopiperazine Derivatives from Actinobacteria

**DOI:** 10.1002/cbic.202200502

**Published:** 2022-10-05

**Authors:** Jing Liu, Shu‐Ming Li

**Affiliations:** ^1^ Institut für Pharmazeutische Biologie und Biotechnologie Fachbereich Pharmazie Philipps-Universität Marburg Robert-Koch-Straße 4 35037 Marburg Germany; ^2^ Current address: Department of Natural Products in Organismic Interactions Max Planck Institute for Terrestrial Microbiology Karl-von-Frisch-Straße 10 35043 Marburg Germany

**Keywords:** 2,5-DKPs, dimeric DKPs, genome mining, heterologous expression, nucleobase-containing DKPs

## Abstract

Secondary metabolites derived from microorganism constitute an important part of natural products. Mining of the microbial genomes revealed a large number of uncharacterized biosynthetic gene clusters, indicating their greater potential to synthetize specialized or secondary metabolites (SMs) than identified by classic fermentation and isolation approaches. Various bioinformatics tools have been developed to analyze and identify such gene clusters, thus accelerating significantly the mining process. Heterologous expression of an individual biosynthetic gene cluster has been proven as an efficient way to activate the genes and identify the encoded metabolites that cannot be detected under normal laboratory cultivation conditions. Herein, we describe a concept of genomics‐guided approach by performing genome mining and heterologous expression to uncover novel CDPS‐derived DKPs and functionally characterize novel tailoring enzymes embedded in the biosynthetic pathways. Recent works focused on the identification of the nucleobase‐related and dimeric DKPs are also presented.

## Graphical abstract

**Genome mining** combined with heterologous expression has been demonstrated to be an efficient way to identify novel 2,5‐diketopiperazine derivatives from actinobacteria. Meanwhile, their biosynthetic pathways and new enzymes embedded inside have been functionally characterized. It is expected that more biologically active diketopiperazines can be obtained by exploring the genetic potentials of microorganism in the future.

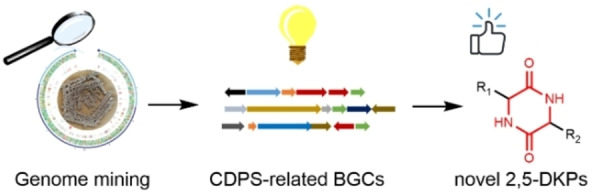

## Natural Products from Microorganisms

Microbes, especially actinobacteria and filamentous fungi, are important and prolific sources of small molecules, also known as natural products (NPs).^[1]^ Many biologically active microorganism‐derived NPs have been widely used and made significant contribution in the fields of medicine and agriculture.^[2]^ Notably, approximately two‐thirds of clinically used antibiotics are natural products or derived thereof.^[3]^ Nowadays, there is an ever‐increasing demand for novel antibiotics and bioactive metabolites because of the emergence and global spread of multidrug‐resistant pathogens.^[4]^ However, owing to a decline of new microbial producers and the frequent re‐discovery of known metabolites, the discovery of new compounds by the traditional bioactivity‐guided isolation strategy is becoming more and more difficult.^[5]^

With the development of the next‐generation sequencing (NGS) technologies, a large number of microbial genome data have been exposed in the public databases with unprecedentedly speed.^[6]^ The microbial genes coding for enzymes that synthesize a specific NP typically span a constituted region on the chromosome and present as a so‐called biosynthetic gene cluster (BGC). Analysis of these genomes revealed the presence of a large number of uncharacterized BGCs, which are silent or incapable to express under standard laboratory conditions in the natural hosts, indicating that the ability of many microorganisms to produce NPs have been far underestimated.^[7]^ For example, in addition to the five well‐known isolated compounds, bioinformatic analysis of the complete genome sequence of the model actinomycete *Streptomyces coelicolor* revealed the presence of 18 additional putative BGCs related to SM production.^[8]^ Therefore, a great set of strategies, such as genome mining and heterologous expression, have been developed to access these cryptic BGCs, thereby exploring the embedded metabolites.^[9]^ It can be expected that the microbial chemo‐diversity increased in this way will play an important role in future for drug discovery and development.

## Genome Mining and Heterologous Expression

**Figure 1:**
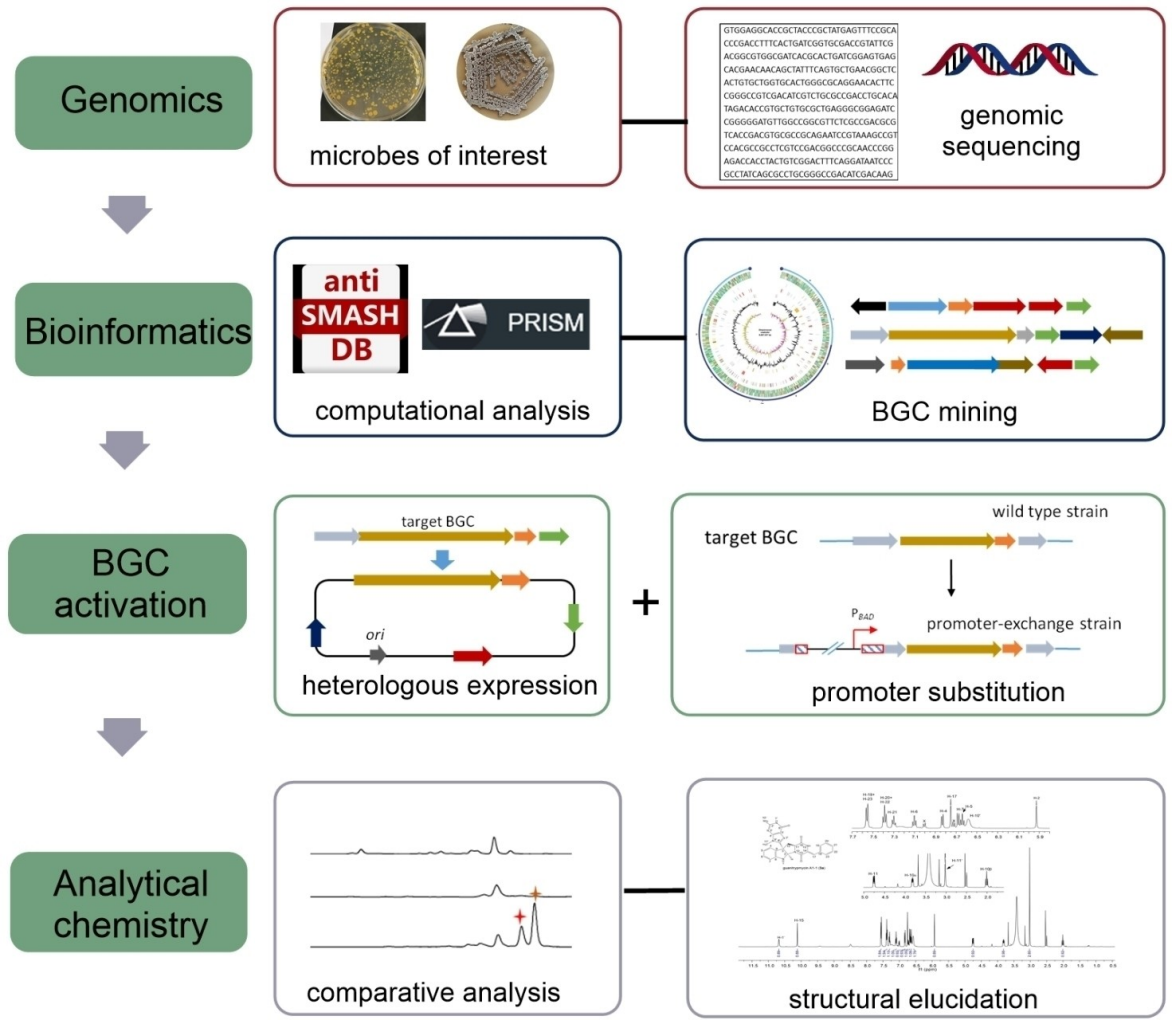
Genomics‐guided natural product discovery.

The concept of genome mining first emerged in the early 2000s and has made tremendous advancements thereafter.^[10]^ The term of genome mining refers to the utilization of genomic information to discover novel products.^[11]^ It involves multiple steps including the initial bioinformatic analysis and identification of unknown BGCs in the genomes of interest, sequence analysis of the coding genes, and identification of the molecules synthesized by these BGCs (Figure 1).[^9b^, ^12^] Among them, the effective prediction and accurate selection of uncharacterized BGCs for novel metabolites should be the highest priority for microbial genome mining process.^[13]^ Several computational tools have been developed to analyze and functionally annotate the genome sequences.^[14]^ One representative tool is the widely used web‐based software “Antibiotics and Secondary Metabolite Analysis SHell” (antiSMASH).^[15]^ This platform provides a rapid identification and detailed functional annotation of BGCs, and even prediction of possible chemical structures of the corresponding pathway products by comparison with the known BGCs. Meanwhile, new biosynthetic pathways and novel enzymes can be predicted and identified. This comprehensive pipeline enable to detect BGCs for a wide variety of metabolites, including polyketides (PKs), non‐ribosomal peptides (NRPs), PKS/NRPS hybrid metabolites, terpenoids, alkaloids, along with ribosomally synthesized and post‐translationally modified peptides (RiPPs). Moreover, antiSMASH achieves very high accuracy for bacterial cluster annotation, which accelerates the mining process and enables the researchers to quickly find out the interested BGC for further study.

PRISM, freely available at http://prism.adapsyn.com, is another representative genome analysis in silico tool.^[16]^ Same as antiSMASH, the predictions performed by PRISM employ a Hidden Markov Model (HMM) of genes that are specific for certain types of BGCs. The old version of PRISM was specialized in predicting the possible chemical structures resulting from NRPSs and PKSs.^[17]^ PRISM4 released in 2020 is capable of prediction for all classes of bacterial natural antibiotics currently in clinical use, including aminoglycosides, nucleosides, β‐lactams, alkaloids, and lincosamides.^[16]^ The other programs like ClusterFinder,^[18]^ BiG‐SCAPE,^[19]^ and NP.Searcher^[20]^ are useful tools to predict specific classes of SMs. Moreover, SMURF is the first web‐based search platform to predict putative backbone genes with high accuracy from fungal genome.^[21]^

A phylogeny‐guided mining strategy makes it possible to quickly target new genes or BGCs of a specific class among a large number of microbial genomes or metagenomes.^[22]^ In the past, phylogenetic trees were commonly used to describe evolutionary relationships among species.^[23]^ More recently, the molecular phylogeny has become a powerful tool for gene or protein sequence comparisons. The function of unknown genes could be predicted based on their phylogenetic position in relation to the known ones.^[24]^ For NP BGCs, the phylogenetic trees are usually built either with the core biosynthetic genes or with tailoring enzymes as the molecular marker.^[22]^

Generally, if the unknown gene is located close to the characterized genes, it is likely that they share similar functions. In contrast, if the unknown sequence falls out of a phylogenetic subclade, it probably codes an novel enzyme for a new reaction.^[22]^ Hence, it allows us to predict a possible chemical structure or product family encoded by the BGC, if we could get a critical amount of data and information.^[24]^

Mining of microbial genomes using in silico tools mentioned above confirmed their great potential to produce specialized metabolites. In recent years, multiple new strategies have been implemented to awake cryptic gene clusters and trigger the corresponding SM overproduction.^[10a]^ Some approaches such as growth condition optimization, co‐cultivation with other microorganisms, and addition of chemical elicitors may induce the expression of more than one BGC.^[25]^ For a special BGC, heterologous expression of the entire BGC in a well‐established host and insertion of constitutive or inducible promoters are the most extensively used methods.^[26]^ Heterologous expression has been proven to be a powerful approach to activate BGCs of interest, thus accessing the special SMs and characterizing their biosynthetic pathways.[^9a^, ^27^] Typically, this approach relies on efficient cloning of intact BGCs onto suitable expression vectors.

The individual BGCs can be captured by screening the genomic libraries constructed based on cosmid, fosmid or bacterial artificial chromosome (BAC).^[9a]^ Alternatively, the candidate BGCs can be directly cloned from genomic DNA. Direct Pathway Cloning (DiPaC) is an efficient method to clone small‐ and mid‐size BGCs. The linear DNA fragments were obtained by PCR and then assembled with the vector via ligation or Gibson assembly method.^[28]^ Methods for large BGC cloning range from linear‐linear homologous recombination (LLHR),^[29]^ ExoCET (Exonuclease Combined with RecET recombination),^[30]^ to yeast‐based transformation‐associated recombination (TAR) cloning approach.^[31]^ A reliable host is also crucial to ensure the successful expression of BGCs.^[32]^ One additional challenge is the availability of necessary building blocks or substrates in the expression host.^[33]^ Therefore, the selected BGCs are commonly expressed in the hosts sharing the similar genetic background as the original holders.^[34]^ In recent years, several microbial strains have been optimized and engineered as the expression hosts. *Streptomyces coelicolor*,^[35]^ *Streptomyces lividans* ^[36]^ and *Streptomyces albus*,^[37]^ are the most widely used strains for efficient expression of BGCs derived from actinobacteria, while *Aspergillus nidulans* ^[38]^ and *Aspergillus oryzae* ^[39]^ are hosts for those with fungal origin. In some cases, the drawback of this approach is that the yields remain stuck at low level. Additional approaches including refactoring of the clusters with promoter exchange and insertion of regulatory regions are therefore performed to modify the expression constructs in order to alter gene expression level.[^29^, ^40^]

## Genomics‐Based Discovery of Novel 2,5‐DKPs

2,5‐Diketopiperazines (2,5‐DKPs) by condensation of two α‐amino acids belong to the smallest cyclic peptides^[41]^ and are characterized by a central DKP core. The 2,5‐DKPs are widely distributed in nature and constitute a large class of bioactive metabolites isolated from microorganisms, plants, and mammals.^[42]^ In the last years, they have gained much more attentions because of their biological and potential pharmacological activities, such as antibacterial, antifungal, antitumor, and immunosuppressive effects.^[43]^ For instance, Plinabulin (formerly known as BPI‐2358), the derivative of phenylahistin, shows potent antitumor activity against a broad spectrum of tumor cell lines and is currently in a world‐wide Phase III clinical trial for non‐small cell lung cancer treatment in combination with docetaxel.^[44]^ Tadalafil, achieved via chemical synthesis, is used to treat male erectile dysfunction (ED).^[45]^

In organism, the 2,5‐DKP scaffolds are biosynthesized by two distinct types of enzymes, i. e., NRPSs and cyclodipeptide synthases (CDPSs). NRPSs are well‐studied large multi‐domain machineries.^[46]^ At least three core domains are found in a typical NRPS module: the adenylation (A) domain, the peptidyl carrier protein (PCP) domain and the condensation (C) domain. One module is responsible for the incorporation of one amino acid as a building block to the polypeptide chain in a catalytic round.^[47]^ Two‐module NRPSs are mainly found in fungi for 2,5‐DKP formation. In comparison to NRPSs, CDPSs are predominantly distributed in bacteria, especially in actinobacteria. CDPSs, consisting of 200–300 amino acid residues, are small proteins of approximate 25–30 kDa.^[48]^ They utilize directly aminoacyl‐tRNAs (aa‐tRNAs) from the primary metabolism as substrates to synthesize the DKP scaffolds.^[49]^ To date, more than 100 CDPSs have been functionally characterized for assemble more than 75 different CDPs.^[50]^

**Figure 2:**
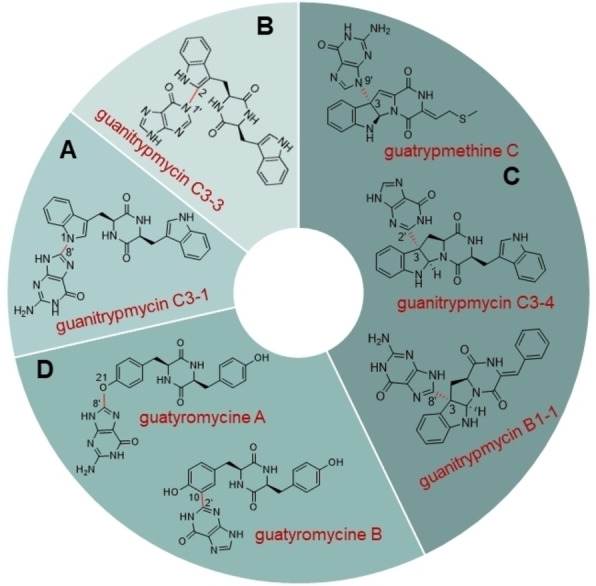
Nucleobase‐containing DKP derivatives identified by genomics‐guided approaches.

Similar to most NPs from microorganisms, genes coding for various classes of tailoring enzymes are located close to the *cdps* genes, which introduce specific modifications to the generated DKP scaffolds for more complex metabolites.[^50^, ^51^] The representative modification enzymes are oxidoreductases including cytochrome P450s (P450s), Fe(II)/α‐ketoglutarate‐dependent oxygenases, cyclodipeptide oxidases (CDOs), different types of transferases such as prenyltransferases (PTs) and methyltransferases (MTs), as well as terpene cyclases.^[51a]^ Very recently, dozens of new DKP‐containing compounds have been identified by elucidation of CDPS‐dependent biosynthetic pathways, particularly of those containing CDPS and P450s, by genome mining and heterologous expression.^[52]^ Featured structures range from nucleobase‐containing DKPs to dimeric tryptophan‐containing CDPs. At the same time, novel P450s that catalyze intriguing chemical transformations like intermolecular C−C bond formation and nucleobase transfer were characterized (Figure 2).^[53]^

## Nucleobase‐Containing DKPs and Derivatives

Represented nucleobase‐containing molecules include DNA and RNA, nucleobase‐related peptides and nucleoside antibiotics.^[54]^ Nucleoside antibiotics, consisting of a saccharide core and a nucleobase, belong to a NP class with wide spectrum of biological activities.^[55]^ Other types of nucleobase‐related NPs are rarely reported prior to our study on CDPS‐related pathways. Unprecedentedly, bioinformatics and phylogenetic analysis of *Streptomyces purpureus* genome revealed the presence of a novel BGC with *cdps* and *p450* genes. Introduction of the whole BGC into *S. coelicolor* resulted in the discovery of 1‐(8‐guaninyl)‐cWW (guanitrypmycin C3‐1) (Figure 2A).[^52a^, ^56^] Meanwhile, the P450 enzyme was identified as the first example that catalyzes the unique C−N bond linkage between the guanine residue and a DKP indole ring. Inspired by this finding, we have focused on the discovery of new DKP derivatives via exploring CDPS‐associated BGCs from actinobacteria by genome mining since 2019. We observed a special five‐gene operon in two *Streptomyces* species, coding for CDPS, P450, CDO, and MT proteins. Heterologous expression of the BGCs resulted in the production of C3‐guaninyl indole alkaloids guanitrypmycins and implied the P450 enzymes GutDs as another new biocatalyst group for the key C−C linkage between DKP and guaninyl moiety (Figure 2C).^[52b]^ The biosynthetic pathways of guanitrypmycins were elucidated via expression of different gene combinations. Further biochemical investigation confirmed that the two cytochrome P450s functioned as key enzymes to catalyze the regio‐ and stereospecific transfer of a guaninyl moiety to C3 of the indole ring. This study represented an excellent example for discovering of novel 2,5‐DKPs by genome mining as well as functional characterization of biosynthetic enzymes.

Interestingly, in addition to the guaninylated DKPs, we also discovered new DKP derivatives with another nucleobase hypoxanthine. Two *cdps‐p450* gene clusters from *Streptomyces lavendulae* and *Streptomyces xanthophaeus* can synthesize hypoxanthine adducts of cWW as main products, whereby the hypoxanthine residue were attached onto C2 and C3 of the indole ring via C−N and C−C linkages, respectively.^[52c]^ Meanwhile, the cWW‐based guaninylated DKPs were also found as the minor products (Figure 2B, 2C).

It should be pointed out that in the aforementioned structures, the nucleobases are attached to indole or indoline rings of DKPs containing tryptophan and a second aromatic acid like cWW, cWF and cWY. Computational search by using the characterized P450 GutD as the probe in the public database (NCBI) and further comparative analysis led to the identification of a cryptic gene cluster in *Streptomyces cinnamoneus* with five genes coding for a CDPS (GtmA), a CDO (GtmBC), a P450 (GtmD) and a Fe(II)/α‐ketoglutarate‐dependent oxygenase (GtmE).^[52e]^ In analogy to our previous studies, the whole BGC was amplified, cloned into the widely used pPWW50A vector and introduced into *S. albus* J1074 for heterologous expression. The end product of this specific gene locus was identified as guatrypmethine C, a guaninylated cWM derivative with two double bonds *exo* the DKP ring (Figure 2C).

In addition to adducts of tryptophan‐containing DKPs, nucleobases can also be attached to tyrosine‐containing cyclodipeptide like cYY. Mining of *Streptomyces* genomes led to the identification of a widely distributed genetic locus (*gym* BGC) with *cdps‐p450* orthologous. Expression of six of these two‐gene BGCs in *S. albus* resulted in the accumulation of mycocyclosin, guatyromycine A with a guaninyl residue attached to the phenolic hydroxyl group and guatyromycine B with a hypoxanthine at its *ortho*‐position (Figure 2D).^[52f]^ The involved cytochrome P450s were identified as the first dual‐functional oxidases from CDPS‐P450‐dependent pathways, which performed as both intramolecular oxidases and intermolecular nucleobase transferases.

## Dimeric DKPs and Derivatives thereof

**Figure 3:**
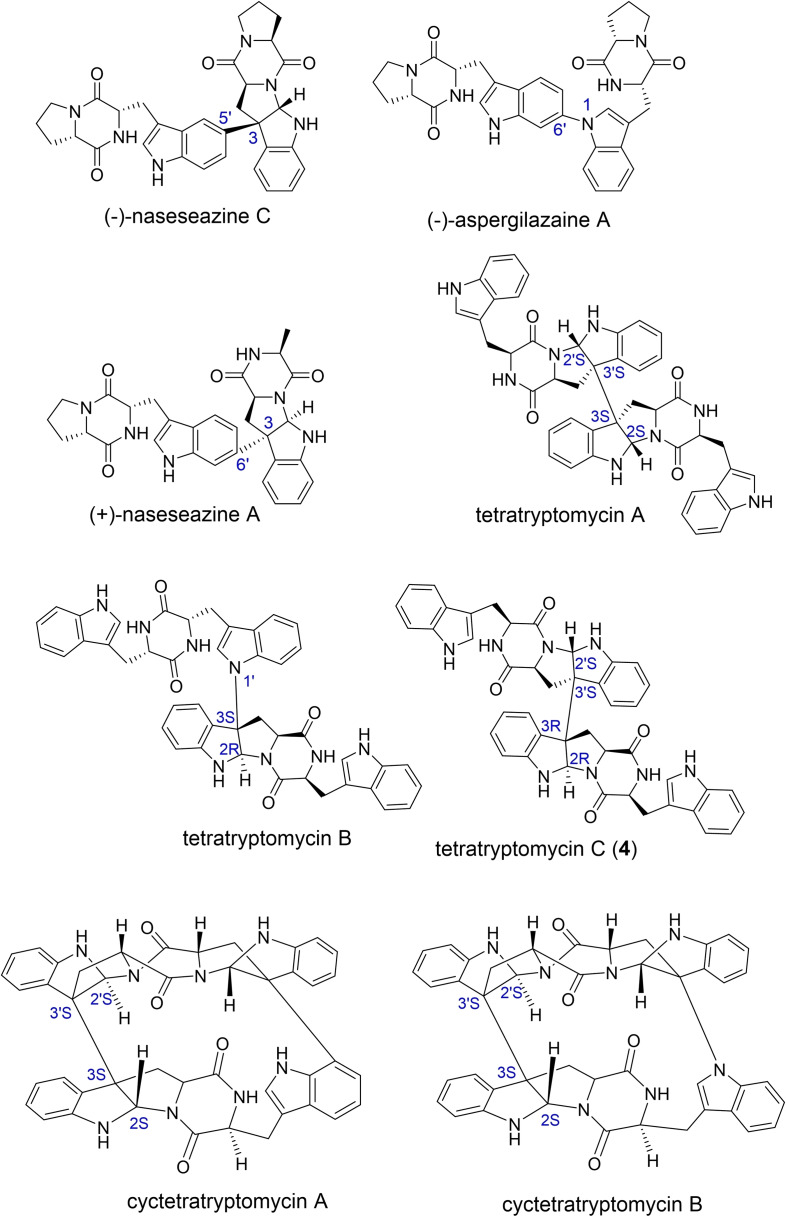
Dimeric DKPs identified by genomics‐guided approaches.

To date, more than 50 tryptophan‐based dimeric DKPs have been isolated from various origins.^[57]^ Due to the dimerization patterns and stereo‐specificities in the dense frameworks, along with the potential and promising pharmaceutical applications, the dimeric DKPs have gathered a lot of interests in recent years.^[58]^ Thus, extensive efforts have been made for their preparation via chemical synthesis, such as (+)‐WIN 64821,^[59]^ (−)‐ditryptophenaline,^[59]^ and (+)‐naseseazines.^[60]^ Alternatively, genome mining together with heterologous expression of *cdps‐p450* BGCs from actinobacteria provided a wide range of non‐symmetrical dimeric DKP natural products with both regio‐ and stereospecificity. Based on the investigation of the biosynthesis of the dimeric DKP naseseazine C (NAS‐C), which features the hexahydropyrrolo[2, 3‐*b*]indole (HPI) framework, the P450 enzyme NascB was characterized as a fixable catalyst with broad spectrum of substrates (Figure 3). Totally, 30 NAS analogs were thus generated from the highly efficient whole‐cell biocatalytic system with involvement of NascB.^[61]^ Interestingly, in some strains, at least two *cdps‐p450* operons can be found in their genomes, which produce distinct dimeric DKPs. For instance, N1−C6′ dimer of two cWP molecules (aspergilazine A) and C3−C6′ connected cWP with cWA (naseseazine A) were identified as the predominant products for two similar *cdps‐p450* BGCs from *Streptomyces* sp. NRRL S‐1868, respectively (Figure 3).^[56]^ Similar phenomenon has also been observed in *Saccharopolyspora antimicrobica*. Dimeric tetratryptomycins with C−C and C−N bond linkages have been identified via refactoring the two BGC into the heterologous host (Figure 3).^[52d]^ These similar loci could come from the common ancestor and acquire adaptive characters after horizontal gene transfer. Similarly, two novel crown‐like CDPs cyctetryptomycins with an unprecedented complex macrocycle were identified by global genome mining of 829 unique cytochrome P450‐associated *cdps*‐containing BGCs (Figure 3).^[62]^

## Perspectives

As mentioned above, various novel 2,5‐DKP derivatives from a large number of CDPS‐related biosynthetic pathways were identified and functionally characterized by genome mining and heterologous expression, which significantly expands the spectrum of DKP diversification. Most of them featured novel tailoring enzymology with new chemical reactions. How to fully utilize these unique biocatalysts or combine them with other enzymes from other types of biosynthetic pathway to create more wonderful compounds, remains unexplored areas. Genomics suggests a large abundance of cryptic CDPS‐associated BGCs waiting for exploration. Skinnider *et al*. developed a pipeline for the identification of CDPS‐associated BGCs and the prediction of the chemical structures of the final DKP products.^[63]^ They used this tool to perform a global analysis of *cdps*‐related BGCs in over 93,000 genomes. Intriguingly, the putative biosynthetic genes coding for N‐acetyltransferases, sulfotransferases, glycosyltransferases and epimerases are found closely associated with CDPSs. These observation indicates that these clusters may code for numerous novel DKPs. Moreover, the ever‐increasing genomic data implied the enormous potential to synthesize numerous secondary metabolites by the human‐associated and environmental microorganisms.^[64]^ To date, we still have a limited understanding of NPs from these two mysterious origins. Efficiently introducing such metagenomic DNA into a laboratory host for heterologous expression could give us more new DKP metabolites. It will in turn allow the investigation of potential ecological interactions between the microbe and human/environments.

## Conflict of interest

The authors declare no conflict of interest.
